# Loss of the Protein Tyrosine Phosphatase PTPN22 Reduces Mannan-Induced Autoimmune Arthritis in SKG Mice

**DOI:** 10.4049/jimmunol.1502656

**Published:** 2016-06-10

**Authors:** Shatakshi Sood, Rebecca J. Brownlie, Celine Garcia, Graeme Cowan, Robert J. Salmond, Shimon Sakaguchi, Rose Zamoyska

**Affiliations:** * Institute of Immunology and Infection Research, Ashworth Laboratories, University of Edinburgh, Edinburgh EH9 3FL, United Kingdom; and; † Experimental Immunology, Immunology Frontier Research Center, Osaka University, Suita 565-0871, Japan

## Abstract

The cytoplasmic phosphatase, protein tyrosine phosphatase nonreceptor type 22 (PTPN22), is a negative regulator of T cell signaling. Genome-wide association studies have shown that single-nucleotide polymorphisms in *PTPN22* confer an increased risk of developing multiple autoimmune diseases in humans. The precise function of PTPN22 and how the variant protein contributes to autoimmunity is not well understood. To address this issue, we investigated the effect of PTPN22 deficiency on disease susceptibility in a mouse model of autoimmune arthritis. The SKG mouse expresses a hypomorphic mutant allele of ZAP70, which, upon exposure to fungal Ags, predisposes the mice to a CD4^+^ T cell–mediated autoimmune arthritis that closely resembles rheumatoid arthritis in humans. Surprisingly, SKG *Ptpn22^−/−^* mice developed less severe mannan-induced arthritis compared with SKG mice. Diminution of disease was not due to significant alterations in thymocyte development or repertoire selection in SKG *Ptpn22^−/−^* mice, even though T cell–mediated signal transduction was improved. Instead, *Ptpn22* deficiency appeared to bias CD4 Th cell differentiation away from the Th17 lineage, which is pathogenic in this setting, to a more Th1/T regulatory–focused response. These data show that even small perturbations in TCR signal transduction pathways can have profound consequences on the differentiation of T cell lineages and thus for the development of autoimmune diseases.

## Introduction

Rheumatoid arthritis (RA) is a chronic systemic inflammatory disease that predominantly affects the synovial membranes of the primary joints. Although autoreactive CD4^+^ T cells and dysregulated B cell homeostasis are prime mediators of RA, the precise etiology remains unknown (1). Recent genome-wide association analysis has identified a single-nucleotide polymorphism, R620W, in the *PTPN22* gene as a predominant risk factor for RA and other autoimmune diseases (reviewed in Ref. 2).

*PTPN22* encodes a phosphatase known as lymphoid tyrosine phosphatase in humans or proline, glutamic acid, serine, threonine–enriched phosphatase in mice. The role of PTPN22 is best characterized in T cells, where it functions to inhibit TCR signaling by dephosphorylating key members of the T cell signaling pathway, including Src family kinases such as Lck, ITAM residues on TCR-ζ and CD3 chains, ZAP70, and Vav (3, 4). *Ptpn22^−/−^* mice show increased TCR signaling particularly in effector cells with an age-dependent increase of effector-memory CD4^+^ and CD8^+^ T cells (5, 6). On a C57BL/6 (B6) background, *Ptpn22^−/−^* mice do not show signs of autoimmunity (5, 6) unless the PTPN22 deletion is combined with another susceptibility mutation such as CD45-E163R (7). PTPN22 R619W knock-in mice, which are analogous to the human R620W variant and show a similar, albeit milder phenotype to that of the *Ptpn22^−/−^* mouse (8, 9), were found to develop multiple features of autoimmunity on a susceptible mixed B6/129 background (8). Collectively, these data indicate that there is synergy between susceptibility alleles that contribute to an autoimmune phenotype.

An association of PTPN22 dysfunction with autoimmunity fits well with the known roles of phosphatases in attenuating signaling pathways integral to immune cellular reactivity (10). However, the underlying molecular mechanism by which PTPN22 regulates the adaptive immune responses to contribute to maintenance or breaking of tolerance is not well understood. Although a number of PTPN22 targets have been identified in vitro, it is unclear which of these are most relevant to the development of autoimmunity. To address this issue, we used the ZAP70^skg/skg^ (hereafter called SKG) mouse that has a defect in ZAP70 kinase activity and expression and develops CD4^+^ T cell–mediated autoimmune arthritis (11). In mice reared under specific pathogen-free conditions, arthritis can be elicited either through activation of innate immunity, by administration of fungal extracts, or by provoking homeostatic proliferation of self-reactive T cells upon transfer to the T cell–deficient environment (12). The SKG mouse has a spontaneous point mutation in ZAP70, resulting in an amino acid change, W163C within the Src homology 2 domain. ZAP70^skg/skg^ homozygous mice have a ∼80% decrease in ZAP70 protein abundance compared with wild-type (WT) BALB/c mice and decreased signaling through the TCR complex (11). Substrate trap experiments show that ZAP70 is one of the substrates of PTPN22, which dephosphorylates the activating tyrosine residue (Tyr^493^) on ZAP70 (4). We generated double-mutant SKG *Ptpn22*-deficient mice (SKG *Ptpn22*^−^*^/^*^−^) by backcrossing SKG mice with mice containing global deletion of *Ptpn22* to ask what influence combining these two mutations would have on the development and progression of arthritic disease.

We show that PTPN22 deficiency enhanced TCR-mediated signaling in SKG *Ptpn22^−/−^* thymocytes and that the early stages of thymus positive selection were partially restored in SKG *Ptpn22^−/−^* mice. However, the selected peripheral TCR repertoire of SKG *Ptpn22^−/−^* mice was not substantially changed from that of SKG mice. Consistent with increased signaling observed in the thymocytes, there were improved in vitro proliferation and IL-2 production of CD4^+^ T lymphocytes from SKG *Ptpn22^−/−^* mice compared with SKG mice. Surprisingly, SKG *Ptpn22^−/−^* mice developed less severe mannan-induced arthritis than SKG mice and showed decreased proportions of Th17 and slightly higher numbers of T regulatory cells (Tregs). Therefore, the absence of PTPN22 partially compensates for the defect in ZAP70 activity in the SKG mouse. The increase in TCR signaling capacity influenced the differentiation outcomes of SKG *Ptpn22^−/−^* Th cells with a corresponding decrease in the incidence of autoimmune disease.

## Materials and Methods

### Mice

*Ptpn22* Exon1^fl/fl^ × PC3-Cre (global *Ptpn22^−/−^*) (5) mice were backcrossed onto the ZAP70*^Skg/Skg^* mouse line (11) for more than eight generations to generate SKG *Ptpn22^−/−^* double-mutant mice. BALB/c *Rag2^−/−^* and BALB/c mice were bred in house. All mouse strains used were maintained and bred under specific opportunistic pathogen-free conditions, at the University of Edinburgh animal facilities, and experiments were conducted in accordance with the United Kingdom Animals (Scientific Procedures) Act, 1986, and local ethically approved guidelines.

### Cell purification

Single-cell suspensions from lymph nodes (LNs) of BALB/c, SKG, and SKG *Ptpn22^−/−^* mice were stained with anti-MHC class II (M5114) and CD8α (53-67.2) rat Abs and enriched by negative selection using sheep anti-rat IgG Dynabeads (Life-Technologies), according to the manufacturer’s instructions. The negatively enriched CD4^+^ T cell population was then sorted on a FACSAria I (BD Biosciences) to obtain pure populations of CD4^+^CD25^−^CD45RB^hi^ naive T cells (>99% purity).

### Flow cytometry and Abs

Cells were resuspended in FACS buffer (PBS containing 1% FCS and 0.05% sodium azide) and incubated with Abs for 30 min on ice, followed by washes in FACS buffer before acquisition on MACSQuant (Miltenyi Biotec) or LSR-II (BD Biosciences) flow cytometers and analyzed using Flow Jo software v9.6 (Tree Star). Fluorophore-conjugated mAbs against the following Ags were from eBioscience: CD3-bio (145-2C11), CD8-PE (53-6.7), CD25-allophycocyanin (PC61.5), CD44-allophycocyanin eFluor 780 (IM7), CD45RB-PE (C363.16A), CD62L-PE (MEL-14), TCR-FITC and -biotin (H57-597), IL-17 allophycocyanin (ebio17B7), Vβ3-FITC (KJ25), Vβ5-PerCP (MR9.4), Vβ8.1/8.2-PE (KJ16), and Vβ11-FITC (CTVB11). The following Abs were purchased from BioLegend: CD4-Pacific Blue and -PerCP (RMA 4-5), CD8-PerCP (53-6.7), CD69-biotin (H1.2F3), and IFN-γ-FITC (XMG1.2). CFSE and Cell Tracer Violet were purchased from Invitrogen. For intracellular staining, LN cells prestained with CD4 Ab were stimulated with 50 ng/ml phorbol 12,13-dibutyrate (PDBu; Sigma-Aldrich) and 0.5 μg/ml ionomycin (Sigma-Aldrich) in the presence of 5 μg/ml brefeldin A (Sigma-Aldrich) for 4 h, stained with Live/Dead (Molecular Probes, Invitrogen), fixed and permeabilized with Cytofix/Cytoperm (BD Biosciences), and stained with cytokines anti–IL-10, anti–IL-17, anti-TNF, and anti–IFN-γ. The data were collected using a MacsQuant (Miltenyi Biotec) flow cytometer and analyzed using FlowJo software v9.6 (Tree Star).

### Western blotting

Cells were lysed in 1% Triton X-100, 0.5% *n*-dodecyl-*b*-d-maltoside, 50 mM Tris-HCl (pH 7.5), 150 mM NaCl, 20 mM EDTA, 1 mM NaF, and 1 mM sodium orthovanadate containing protease inhibitors, and samples were separated by SDS-PAGE. Membranes were incubated in blocking reagent (LI-COR Biosciences) before immunoblotting with Abs to ZAP70 and β-actin. Proteins were detected with secondary Abs and visualized using an infrared imaging system (Odyssey; LI-COR Biosciences).

### Calcium flux

Single thymocyte cell suspension, 1 × 10^7^ cells/ml in prewarmed PBS, from BALB/c, SKG, and SKG *Ptpn22^−/−^* mice were labeled with varying concentration of CFDA-SE dye (Life Technologies) for 10 min, protected from light at 37°C. The individual populations were mixed in a 1:1:1 ratio at final cell concentration of 3 × 10^7^ cells/ml and incubated with 2 μM solution of the ratiometric indo-1-AM dye (Life Technologies) for 40 min at 37°C in prewarmed PBS, before washing and staining with 10 μg/ml biotinylated anti-TCR (H57-597), anti-CD3 (145-2C11), and anti-CD4 (RMA 4.5) mAbs. Anti–CD8α-PerCP (53-6.7) and anti–CD4-allophycocyanin (RMA4.4) Abs were used to identify the thymus populations. After the surface staining, cells were resuspended in prewarmed IMDM (Life Technologies) containing 1% FCS and supplemented with 2.5 mM CaCl_2_ and 2.8 mM MgCl_2_. The baseline Ca^2+^ levels were measured for 45 s before cross-linking the biotinylated Abs with 1 μg/ml streptavidin–PE conjugates, and samples were collected on a flow cytometer (LSR II; BD Biosciences). The increase in Ca^2+^ was measured as the ratio of Indo-1-violet to Indo-1-blue fluorescence and displayed as function of time.

### TCR repertoire analysis

mRNA was purified from 10^6^ naive CD4 T cells using a mRNA direct purification kit (ThermoFisher Scientific), and cDNA was synthesized using SMARTscribe reverse transcriptase (Clontech Laboratories) to produce bead-coupled mRNA libraries. The entire volume of mRNA beads was added to 4 μL first-strand buffer, 0.5 μL 20 mM DTT, 2 μL 10 mM dNTP mix, 2 μL SMART synthesis oligo (5′-GGCGAAGCAGTGGTATCAACGCAGAGTACGCrGrGrG-3′), 0.5 μL RNase inhibitor, and 2 μl SMARTscribe reverse transcriptase (Clontech), and the reaction was incubated at 42°C for 1 h.

TCR-β V-region amplicons were generated from cDNA by PCR using indexed forward primers composed of the SMART synthesis oligo sequence fused to a P7 illumina tag, and a reverse primer within the TCR-β C region fused to a P5 illumina tag. Amplified products were purified by extraction from excised agarose gel bands and concentrated by ethanol precipitation prior to sequencing. Single-end 1 × 400-bp sequencing was performed on an Illumina MiSeq sequencer using custom read1 primers and indexing primers complementary to the amplification primer sequences. Sequence data were processed using the MiTCR utility (13) and a customized pipeline of python scripts to analyze and plot the MiTCR output.

### Cell cultures and assays

Cells were cultured in IMDM (Sigma-Aldrich) supplemented with 5% heat-inactivated FBS (Serotec), 2 × 10^−3^ M l-glutamine, 100 U/ml penicillin, 100 μg/ml streptomycin, and 5 × 10^−5^ M 2-ME (Sigma-Aldrich). Sorted naive CD4^+^ T cells were labeled with 2.5 μM Cell Trace Violet (Molecular Probes, Life Technologies) in prewarmed PBS at 37°C for 20 min in dark and washed, and 1 × 10^5^ Cell Trace–labeled T cells were incubated with 2 × 10^5^ irradiated BALB/c *Rag2*^−^*^/^*^−^ splenocytes in presence of 0.04, 0.2, and 1 μg/ml anti-mouse CD3ε Abs (eBioscience) in round-bottomed 96-well plate at 37°C. Cells were harvested at 72 h after stimulation and stained with anti-CD4 and anti-CD8, and proliferation was assessed and analyzed by flow cytometry. Supernatants from stimulated LN cultures were analyzed for IL-2 by ELISA with paired capture and detection Ab from eBioscience. Plates (NUNC Maxisorp) were washed with 0.05% Tween 20 in PBS and blocked with blocking buffer (supplied with kit). Supernatant samples were analyzed in triplicates. After the addition of 100 μL 3,3′, 5,5′ tetramethylbenzidine substrate solution (supplied with kit) and 100 μL 0.18 M H_2_SO_4_ (Carl Roth), absorbance was read at 450 nm with a Laboratory Systems Multiscan Ascent plate reader.

For in vitro Th17 induction, 5 × 10^5^ sorted naive CD4^+^ T cells from LNs were in cultured 1 ml media in a 48-well plate and stimulated with immobilized anti-CD3 (1.5 μg/ml) and anti-CD28 (3 μg/ml) in the presence of a mixture of the following cytokines: TGF-β (1 ng/ml), IL-1β (10 ng/ml; PeproTech), IL-23 (10 ng/ml; R&D Systems), FICZ (300 nM; Enzo Life Science), IL-6 (20, 2, 0.2, 0.02 ng/ml; eBioscience), anti–IFN-γ (5 μg/ml R4-6A2; eBioscience), anti–IL-2 (JES 6-1A12; BioLegend), and anti–IL-12 and anti–IL-4 (from BD Pharmingen) for 4 d. At the end of the culture, the cells were harvested and restimulated with PDBu and ionomycin before intracellular staining and analysis by flow cytometry.

Peritoneal exudate cells from unimmunized mice were adhered to plastic for 4 h and washed before adding mannan. Twenty-four hours later, supernatants were harvested, and the remaining cells were lysed for analysis of protein content. IL-6 was assayed using an ELISA kit (eBioscience), and the IL-6 concentrations obtained from a standard curve were normalized to the protein values per well.

For the in vivo Treg suppression assay, FACS-sorted naive BALB/c CD90.1^+^CD4^+^CD25^−^CD45RB^hi^ cells (4 × 10^5^) were injected alone or together with FACS-sorted SKG or SKG *Ptpn22*^−^*^/^*^−^ CD45.2^+^CD4^+^CD25^+^ Tregs (2 × 10^5^) i.v. into BALB/c *Rag2*^−^*^/^*^−^ recipients in a 2:1 ratio, as indicated (*n* = 6 mice per group). After 12 d, LNs were assessed by flow cytometry for their proportions of CD90.1^+^Foxp3^−^ naive cells and CD90.1^−^Foxp3^+^ Tregs.

### Induction and scoring of arthritis

Six- to eight-week-old mice were injected once i.p. with 50–75 mg mannan reconstituted in sterile PBS. Mice were weighed and observed for clinical signs of arthritis weekly for 4 wk. Signs of arthritis were assessed by visual scoring where 0 = no joint swelling, 1 = mild to moderate swelling, 2 = substantial swelling of wrist or ankle, and 3 = severe swelling of wrist or ankle. Scores for all the wrists and ankles were combined for each mouse.

## Results

### Absence of Ptpn22 does not alter selection in SKG thymocytes

In SKG mice, decreased signaling results in defective positive and negative thymic selection (11). In contrast, *Ptpn22* deficiency has little apparent influence on thymus selection in B6 mice (5, 6). However, in the context of *Ptpn22* deficiency, there was a report of a slight increase in selection of CD4 cells, but no change in negative selection on a TCR transgenic background (6, 7). Therefore, we asked whether loss of PTPN22 would improve selection of thymocytes on the SKG background.

**FIGURE 1.**
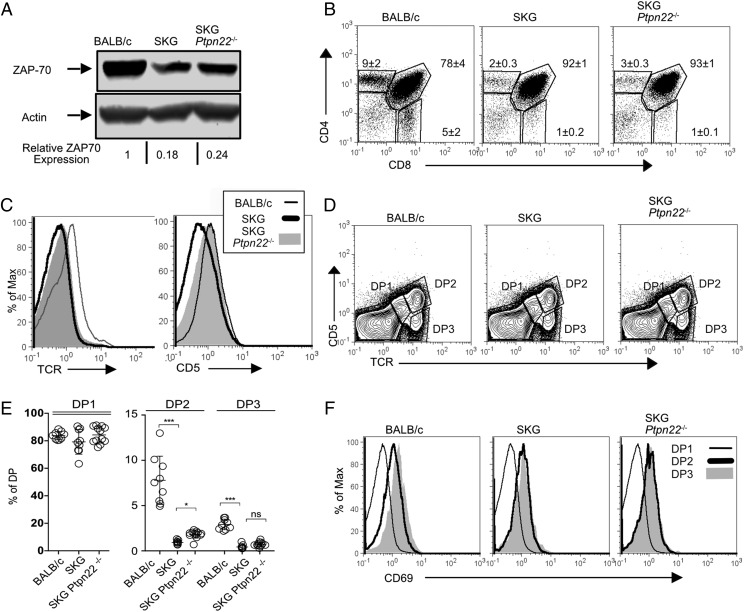
The absence of Ptpn22 does not alter selection in SKG thymocytes. (**A**) The abundance of ZAP70 protein in thymocytes from age-matched (7-wk) BALB/c, SKG, and SKG *Ptpn22*^−^*^/^*^−^ mice was assessed by Western blotting of total cell lysates with ZAP70-specific Ab and β-actin Ab as loading control. Relative expression of ZAP70 was calculated by normalizing the fluorescence of bands to WT BALB/c band. (**B**) *Ptpn22*^−^*^/^*^−^ mice were assessed for the proportions of double-negative, DP, CD4 SP, and CD8 SP thymocytes by flow cytometry. Numbers on the plots denote the averages ± SD of a minimum of three individual mice per group. Data are representative of four independent experiments. (**C**) Histograms show surface expression of αβ-TCR and CD5, as indicated, on gated DP thymocytes. (**D**) Staining of thymocytes with Abs to CD5 and TCR-β was used to distinguish the stages of DP maturation: DP1 (TCR^lo^CD5^lo^), DP2 (TCR^int^CD5^int^), and DP3 (TCR^hi^CD5^int^), as indicated. (**E**) Graphs representing the proportion of thymocyte subpopulation in the DP1, DP2, and DP3 gate. Data represent an average of six to nine mice per genotype (±SD) from three independent experiments. Statistical significance calculated with one-way ANOVA and post hoc Holm–Sidak test for multiple comparisons. *p < 0.01, ***p < 0.0001. (**F**) FACS histogram plots show progressive upregulation of CD69 on DP1, DP2, and DP3 thymocytes from the indicated mouse strains.

SKG mice have a ∼4-fold decrease in the abundance of ZAP70 protein (11). The absence of PTPN22 did not alter ZAP70 protein abundance with SKG *Ptpn22*^−^*^/^*^−^ thymocytes having a similar reduction compared with BALB/c mice (Fig. 1A). SKG thymocytes develop normally until the double-positive (DP) stage (11) in keeping with a limited role for ZAP70 in earlier stages of thymus differentiation (14). Consistent with this, analysis of thymocytes from SKG and SKG *Ptpn22*^−^*^/^*^−^ mice showed that loss of PTPN22 had little effect on the proportions (Fig. 1B) and absolute numbers (Table I) of double-negative and DP thymocytes between these two strains, although total thymocyte numbers for both were reduced relative to control thymocytes from BALB/c mice (11).

**Table I.** Comparison of thymocyte subpopulation numbers in BALB/c, SKG, and SKG *Ptpn22*^−/−^ mice

Genotype	Total	DN	DP	CD4 SP	CD8 SP
BALB/c (*n* = 9)	125.2 ± 13.8	7.0 ± 2.7	98.1 ± 8.8	3.2 ± 1.0	1.9 ± 0.9
SKG (*n* = 9)	113.3 ± 3.2	4.7 ± 1.5	102.5 ± 3.5	0.5 ± 0.1	0.06 ± 0.04
SKG *Ptpn22*^−/−^ (*n* = 12)	112.1 ± 10.4	4.7 ± 3.0	100.8 ± 9.6	0.7 ± 0.3	0.1 ± 0.07

Thymocytes were obtained from 7-wk-old age-matched mice, and the absolute number within each subpopulation was calculated based on the expression of the markers CD4 and CD8 using the gating strategy shown in Fig. 1B.

DN, double negative.

In SKG mice, impaired ZAP70 expression, and therefore function, leads to a failure of both positive and negative selection (11). There was a major reduction in the proportion of TCR^high^ DP thymocytes in SKG mice (11), and we saw an equivalent reduction in the SKG TCR^high^ DP subpopulation in the absence of PTPN22 (Fig. 1C). SKG DP thymocytes have lower CD5 expression than BALB/c DPs, whereas levels of CD5 expression on SKG *Ptpn22*^−^*^/^*^−^ and BALB/c DP thymocytes were similar (Fig. 1C). The higher expression of CD5 on DP thymocytes in the absence of PTPN22 was consistent with reports from *Ptpn22*^−^*^/^*^−^ mice on the C57BL/6 background (5, 6); however, the upregulation of CD5 at this stage did not seem to impact on subsequent selection events. Detailed analysis of the stages of positive selection can be identified by gating DP thymocytes on CD5 and TCR expression to reveal three stages of maturation, as follows: DP1 (CD5^−^TCR^−^), DP2 (CD5^int^ TCR^int^), and DP3 (CD5^int^ TCR^hi^) (Fig. 1D) (15, 16). The abundance of both CD5 and TCR depends on the levels and function of ZAP70 (17), and consequently SKG mice have a lower proportion of DP2 and DP3 cells compared with BALB/c mice, whereas the proportions of DP2 and DP3 subsets were slightly elevated in SKG *Ptpn22*^−^*^/^*^−^ mice compared with SKG mice (Fig. 1E). Selection signals can also be monitored by CD69 upregulation. In BALB/c thymocytes, CD69 expression increased progressively as the cells transited from DP1 to DP3, consistent with these subpopulations becoming positively selected. Although both SKG and SKG *Ptpn22*^−^*^/^*^−^ thymocytes upregulated CD69 between DP1 and DP2 subsets, thereafter CD69 expression was unchanged (Fig. 1F). These data suggest that selection signals at this later stage in the SKG background strains of mice are not maintained to the same extent as they are in BALB/c mice. Indeed, in both SKG and SKG *Ptpn22*^−^*^/^*^−^ thymuses, there were significantly fewer mature CD4 SP and CD8 SP cells generated than in BALB/c mice (Table I), consistent with the impaired positive selection described previously for SKG mice (11). However, there was no significant change in the overall numbers of positively selected thymocytes between SKG and SKG *Ptpn22*^−^*^/^*^−^ mice.

**FIGURE 2.**
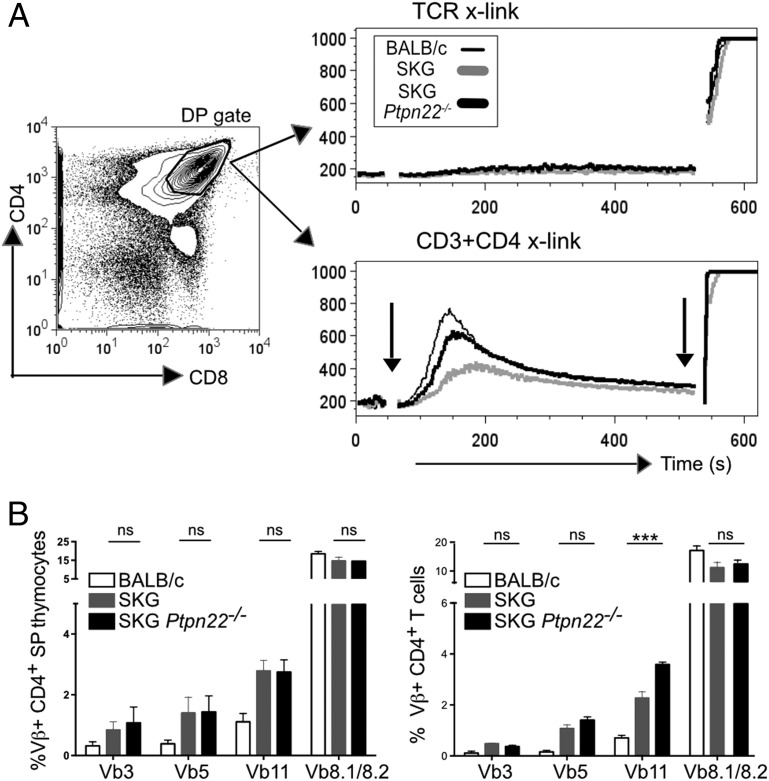
Thymocytes from SKG *Ptpn22*^−^*^/^*^−^ show enhanced TCR-induced calcium mobilization compared with SKG mice. (**A**) Single suspension of BALB/c, SKG, and SKG *Ptpn22*^−^*^/^*^−^ thymocytes was labeled with different concentration of CFSE: 0, 0.012, and 0.002 μM, respectively, mixed in 1:1:1 ratio, and loaded with 2 μM indo-1 dye. Samples were stained with directly conjugated Abs to CD4 (epitope A) and CD8, followed by biotin-conjugated Abs to either TCR-β or CD3 + CD4 (epitope B). Baseline was established for 45 s on gated DP cells, as indicated, and induction of Ca^2+^ flux was achieved by addition of cross-linking streptavidin–PE conjugates. Arrows on the histogram indicate the time of addition of streptavidin (45 s) and ionomycin (8 min) as positive control. Data are representative of three independent experiments. (**B**) Graphs show expression of TCR Vβ3, Vβ5, Vβ11, and Vβ8 alleles in gated mature CD4^+^CD5^hi^ thymocytes (*left*) and peripheral CD4^+^ T cells (*right*) for the individual mouse strains, as indicated. The data are representative of three independent experiments and are shown as averages of *n* = 4 for each strain ± SD. Statistical significance was calculated by one-way ANOVA and post hoc Holm–Sidak test for multiple comparisons. ***p < 0.0001.

Given that ZAP70 has been reported to be a target of PTPN22, we asked whether we could detect differences in signaling through the TCR between SKG and SKG *Ptpn22*^−^*^/^*^−^ DP thymocytes. To control the timing of the delivery of the TCR signal between the individual samples, thymocytes from BALB/c, SKG, and SKG *Ptpn22*^−^*^/^*^−^ mice were labeled with different amounts of CFSE so that they could be mixed together in a 1:1:1 ratio and yet distinguished by FACS. Cells were stimulated by incubating with biotinylated Abs that were cross-linked by addition of streptavidin. Cross-linking CD3 with CD4, but not TCR alone, induced a robust peak of intracellular Ca^2+^ flux in WT BALB/c DP thymocytes, whereas Ca^2+^ flux in SKG thymocytes was impaired, as has been reported previously (11) (Fig. 2A). The absence of PTPN22 partially restored the ability of CD3 plus CD4 cross-linking to induce a Ca^2+^ flux in the SKG DP cells, although it remained lower than WT BALB/c control (Fig. 2A).

In BALB/c mice, thymocytes expressing TCR Vβ3, Vβ5, Vβ11, and Vβ12 chains are reactive with endogenous viral superantigens encoded by Mtv-8 and Mtv-9, and are consequently deleted (18). A graded decrease in TCR signal strength by the expression of aberrant ZAP70 was shown to lead to failure of deletion of self-reactive autoimmune clones in the thymus and thus to alter the peripheral TCR repertoire (19). SKG mice were shown to have an increased frequency of thymocytes bearing these TCR Vβ subfamilies, indicating a failure of negative selection (20). Given that lack of PTPN22 partially restored Ca^2+^ signaling in DP thymocytes, and the strength of Ca^2+^ signaling is directly linked with induction of negative selection at this stage (21), we sought to determine whether there was any effect of *Ptpn22* deficiency on deletion of these particular TCR Vβ families. As described previously (18), the frequency of mature CD4^+^ single-positive (SP) thymocytes and CD4^+^ peripheral T cells expressing TCR Vβ3, Vβ5, or Vβ11 chains was increased in SKG as compared with BALB/c mice (Fig. 2B). Additionally, there was a compensatory decrease in the proportion of TCR Vβ8.1/8.2^+^ cells in SKG mice (20). Loss of PTPN22 did not impact on this failure of negative selection on the SKG background, as there was no significant difference in the proportions of these specific TCR Vβ-expressing cells in either the thymus or periphery of SKG *Ptpn22*^−^*^/^*^−^ compared with SKG mice (Fig. 2B). This result agrees with previous studies reporting that PTPN22 has minimal role in negative selection (6). Overall, these data indicate that PTPN22 deficiency does not impact substantially on either positive or negative selection in the thymus of SKG background mice. We observed a small, but significant increase in Vβ11-expressing T cells in the periphery of SKG *Ptpn22*^−^*^/^*^−^ compared with SKG mice, which was not apparent in the thymus, and it may be that exposure to viral superantigens in the periphery causes greater expansion of this Vβ subset in the absence of PTPN22.

### Similar TCR repertoire but increased responsiveness of SKG Ptpn22^−/−^ CD4^+^ T cells

**FIGURE 3.**
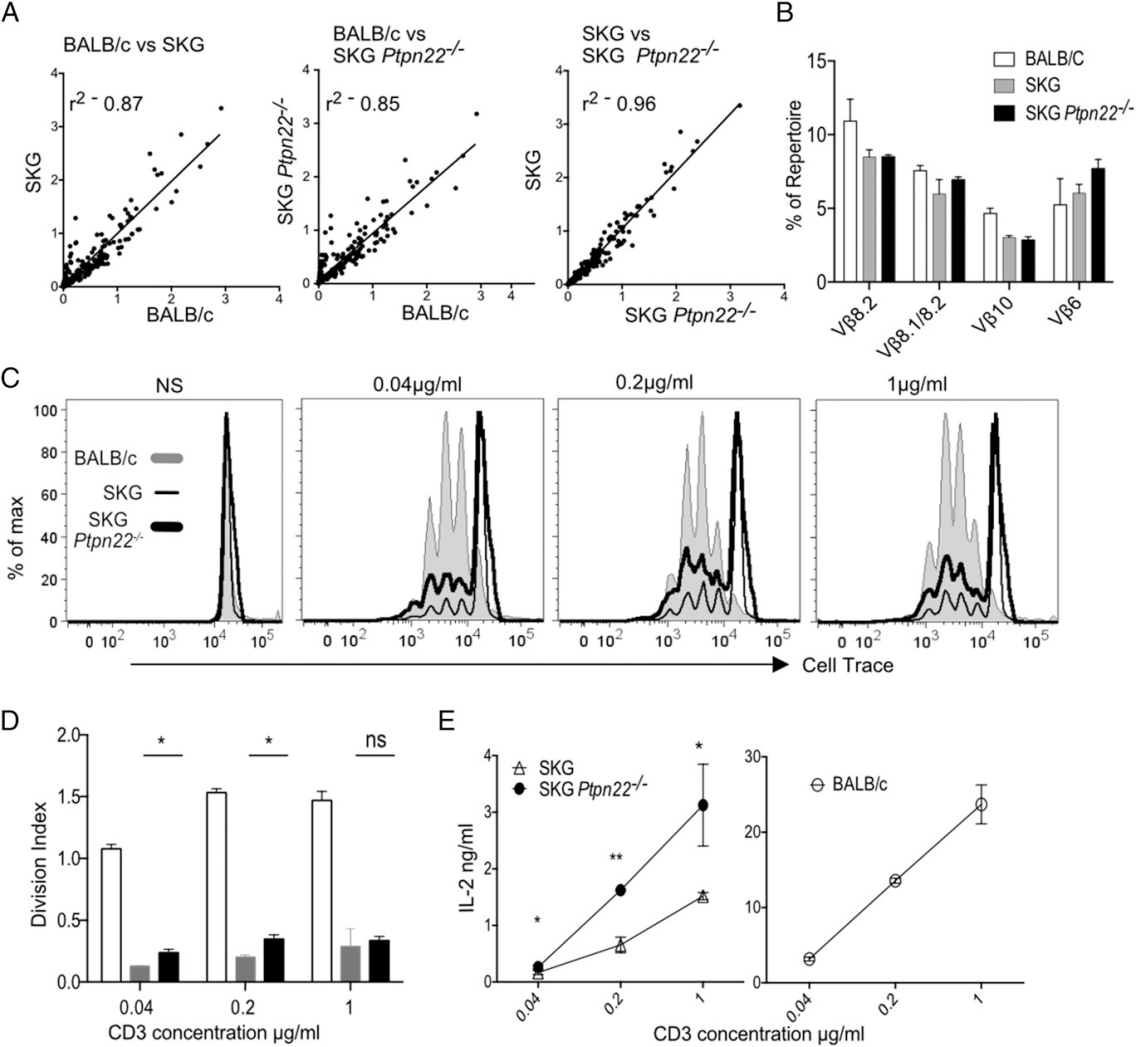
Similar TCR repertoires, but enhanced responsiveness of SKG *Ptpn22*^−^*^/^*^−^ peripheral CD4 T cells. (**A**) Linear scatterplots comparing the frequency of each V/J allelic combination between each pair of mouse genotypes are shown. Best-fit straight lines are plotted for each pairwise comparison, and the corresponding value for goodness-of-fit (*r*^2^) is indicated. (**B**) The frequency of individual Vβ-containing sequences expressed as a percentage of the total repertoire is shown for alleles that have been reported to induce arthritis in SKG mice. (**C**) Sorted naive CD4^+^ T cells (CD25^−^CD45RB^hi^) from BALB/c, SKG, and SKG *Ptpn22*^−^*^/^*^−^ mice (10^5^ cells/well) were stimulated with anti-CD3 and anti-CD28 (2 μg/ml) for 72 h. Proliferation was measured by dilution of Cell-Tracer violet, as shown in the histograms, and (**D**) division index was calculated using FlowJo software. Data representative of four independent experiments. (**E**) Supernatants were harvested and assayed for IL-2 by ELISA. Results are presented as mean ± SD of triplicate samples and are representative of three independent experiments. Statistical significance was calculated using two-way ANOVA; ns, nonsignificant; *p < 0.05, **p < 0.001.

To address more specifically whether the peripheral repertoire was similar between SKG and SKG *Ptpn22*^−^*^/^*^−^ mice, we sequenced their TCR-β CDR3 regions. Naive CD4^+^ T cells were sorted from LN of naive BALB/c, SKG, and SKG *Ptpn22*^−^*^/^*^−^ mice, and TCR-β CDR3 sequences were obtained by next generation sequencing. Linear regression analysis of the TCR Vβ repertoires between BALB/c and SKG or BALB/c and SKG *Ptpn22*^−^*^/^*^−^ mice showed there were considerable differences in VJ usage, indicated by the poor *r*^2^ values, 0.87 and 0.85, respectively, in comparison with that between SKG and SKG *Ptpn22*^−^*^/^*^−^ T cells, which were highly correlated, *r*^2^ = 0.96 (Fig. 3A). More detailed analysis represented by heatmaps of V versus J segment usage showed that there were some subtle changes in VJ usage between the SKG and SKG *Ptpn22*^−^*^/^*^−^ populations (Supplemental Fig. 1A). However, these differences were found to be due to a few VJ combinations that were represented at very low frequencies in SKG or BALB/c repertoires, and were slightly increased in SKG *Ptpn22*^−^*^/^*^−^ mice (Supplemental Fig. 1B). It had been shown previously (22) that arthritogenic T cells in the SKG model were found among TCRs expressing Vβ6, Vβ8.1/8.2, and Vβ10 gene segments. We examined the representation of these specific Vβ alleles among the TCR sequence data and found these Vβs to be at least equally, if not slightly more represented in the absence of PTPN22 (Fig. 3B). Overall, the repertoires of SKG and SKG *Ptpn22*^−^*^/^*^−^ naive CD4 T cells were highly similar, and quite distinct from that of BALB/c mice, further confirming that loss of PTPN22 had little effect on the selection of the naive SKG TCR repertoire.

We asked whether *Ptpn22* deficiency in SKG T cells was able to compensate for the reduced expression of ZAP70 and improve their response to TCR stimulation. Naive CD4^+^ T cells, gated as CD45RB^hi^CD25^−^, were sorted from LNs of young (6-wk-old) BALB/c, SKG, and SKG *Ptpn22*^−^*^/^*^−^ mice, and proliferation was measured following stimulation with anti-CD3 Ab and irradiated splenocytes from BALB/c *Rag2*^−^*^/^*^−^ mice as APC. As previously reported, there was decreased proliferation of SKG CD4^+^ cells compared with that of WT BALB/c in response to all doses of anti-CD3 after 72 h of stimulation in vitro (Fig. 3C, 3D) (11). Although the proliferation of SKG *Ptpn22*^−^*^/^*^−^ cells was reduced compared with BALB/c T cells, SKG *Ptpn22*^−^*^/^*^−^ cells proliferated significantly more than SKG cells, particularly at lower concentration of anti-CD3, as indicated by the division index (Fig. 3C, 3D). In addition, we measured IL-2 cytokine production following stimulation. Although considerably less than produced by WT BALB/c T cells (compare different axes between SKG and BALB/c), SKG *Ptpn22*^−^*^/^*^−^ cells produced more IL-2 than SKG T cells at all anti-CD3 concentrations (Fig. 3E).

### Ptpn22 deficiency decreases the incidence and severity of arthritis in SKG mice

The frequency and clinical score of spontaneous arthritis in SKG mice are dependent on the cleanliness of the environment and microbial colonization. In our animal facilities, SKG mice up to the age of 6 mo did not develop any spontaneous arthritis or any systemic inflammation such as interstitial pneumonitis, as previously reported (23), and loss of PTPN22 did not make them any more susceptible to spontaneous disease up to ∼8 mo of age.

**FIGURE 4.**
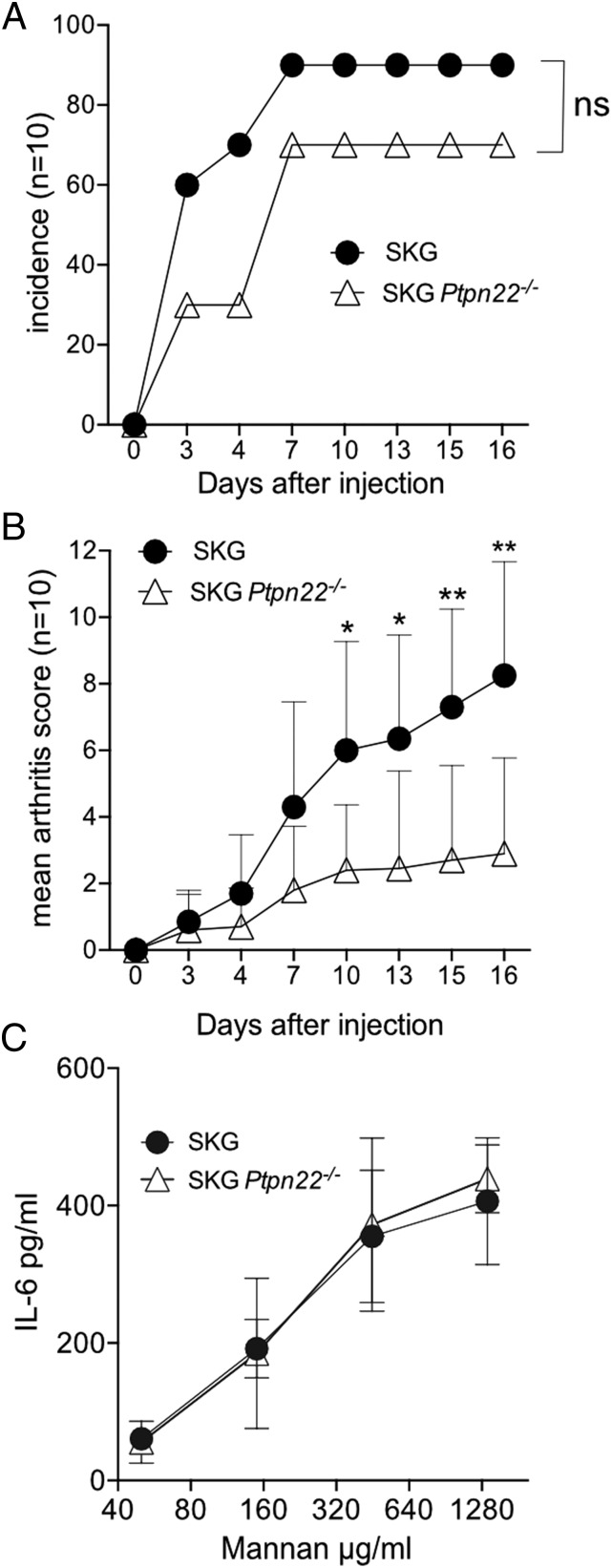
Milder mannan-induced arthritis in SKG *Ptpn22*^−^*^/^*^−^ mice. (**A**) Incidence of arthritis and (**B**) joint scores from 11- to 12-wk-old female SKG and SKG *Ptpn22*^−^*^/^*^−^ mice that received 50 mg mannan i.p. Data representative of two independent experiments. Error bars are mean ± SD of the arthritis scores. Statistical significance for the mean arthritis score was calculated using a paired *t* test; ns, nonsignificant; *p < 0.05, **p < 0.001, and Mantel Cox test was used to assess the significance of arthritis incidence. (**C**) Peritoneal lavage macrophages were adhered to plastic and stimulated overnight with the indicated concentrations of mannan. Supernatants were harvested at 24 h and assayed for IL-6 production by ELISA. Data are the means ± SD of duplicate samples from two biological replicates, and represent one of two independent experiments.

It has been shown that autoimmune disease can be triggered by a single i.p. injection of fungal extracts, such as zymosan, a crude yeast wall extract (12), or mannan, a fungal wall polysaccharide (24). We were unable to elicit any signs of arthritis with the published doses of mannan (0.2, 2, and 20 mg) (25), possibly because, rather than being specific pathogen free (SPF), our mice are of specific opportunistic pathogen-free health status, which may impact on the generation of Th17 T cells (26, 27). Therefore, we used a higher dose of mannan of 50 mg/mouse to provoke disease. A single i.p. injection of 50 mg mannan triggered joint inflammation in both SKG and SKG *Ptpn22*^−^*^/^*^−^ mice. Contrary to expectation, *Ptpn22* deficiency delayed the onset and severity of arthritis in SKG mice. Moreover, the incidence of disease was decreased in SKG *Ptpn22*^−^*^/^*^−^ mice with 3 of the 10 SKG *Ptpn22*^−^*^/^*^−^ mice failing to develop any symptoms of arthritis, compared with only 1 of 10 SKG mice (Fig. 4A). Arthritic joints from mannan-treated SKG and SKG *Ptpn22*^−^*^/^*^−^ mice with comparable arthritis scores showed similar levels of cellular infiltrates (data not shown); however, loss of PTPN22 decreased both the likelihood of getting disease and the overall severity of the resulting arthritis (Fig. 4B).

IL-6 is a key cytokine for the development of arthritogenic Th17 cells in SKG mice, and mannan stimulates IL-6 production by macrophages. We tested whether there was a difference in IL-6 production by SKG or SKG *Ptpn22*^−^*^/^*^−^ peritoneal macrophages after exposure to a range of mannan concentrations, and found the response to be equivalent for both genotypes (Fig. 4C). It is unlikely, therefore, that differences in innate cell responses to mannan were responsible for the resistance to arthritis in the SKG *Ptpn22*^−^*^/^*^−^ background.

### Loss of PTPN22 does not affect Treg numbers or function on the SKG background

We and others (5, 28) have previously reported that *Ptpn22*^−^*^/^*^−^ mice on the B6 background have an increase in Treg numbers. Furthermore, on a per cell basis, *Ptpn22*^−^*^/^*^−^ Tregs were more potent than WT Tregs and suppressed colitis induced by *Ptpn22*^−^*^/^*^−^ effector cells, which WT Tregs were unable to control (5). We examined whether increased Treg production or efficacy accounted for the reduced arthritis found in the SKG *Ptpn22*^−^*^/^*^−^ mice.

**FIGURE 5.**
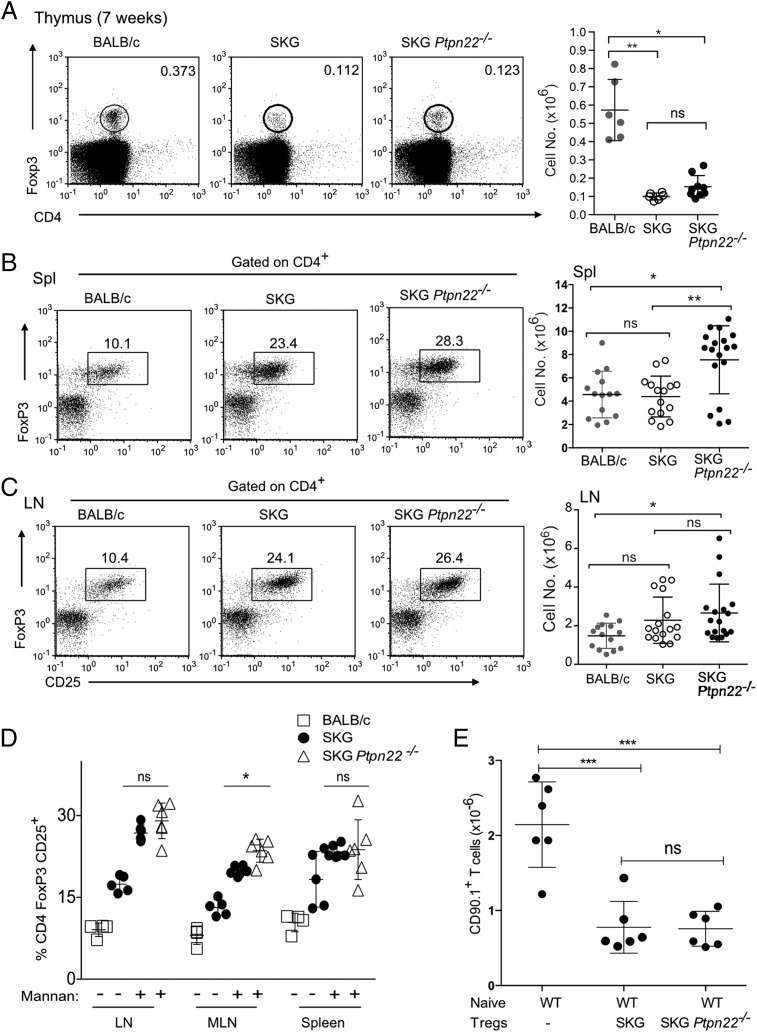
Treg numbers are similar in SKG and SKG *Ptpn22*^−^*^/^*^−^ mice. (**A**) Representative FACS plots and total cell numbers of Tregs present in the thymus of 7-wk-old mice show no difference between SKG and SKG *Ptpn22*^−^*^/^*^−^ mice. (**B**) Older mice, >21 wk old, on the SKG background show expansion of Tregs in the spleen compared with BALB/c mice, and there was a statistically significant increase in absolute numbers of Tregs between SKG and SKG *Ptpn22*^−^*^/^*^−^ mice. (**C**) In contrast, LN Treg proportions and numbers are equivalent in SKG and SKG *Ptpn22*^−^*^/^*^−^ mice, although raised in comparison with BALB/c mice. Each symbol in (A-C) represents an individual of six to nine mice total, and bars represent SD. Statistical significance was calculated using a Kruskal–Wallis test with Dunn’s posttest, adjusted for multiple comparisons. (**D**) Proportions of Tregs were expanded to a similar extent in SKG, and SKG *Ptpn22*^−^*^/^*^−^ mice 3 wk following mannan treatment, with only MLN, showed a slight but significant increase of Tregs in the absence of PTPN22. Each symbol represents an individual of six mice total per genotype (±SD) from two independent experiments. (**E**) In vivo Treg suppression assay. WT sorted BALB/c naive CD90.1^+^CD4^+^ cells (4 × 10^5^) were injected alone or together with SKG or SKG *Ptpn22*^−^*^/^*^−^ CD45.2^+^CD4^+^CD25^+^ Tregs (2 × 10^5^) into BALB/c *Rag2*^−^*^/^*^−^ recipients (*n* = 6 mice per group). After 12 d, recovery of CD90.1^+^Foxp3^−^ T cells was determined. Each symbol represents individual mouse. Bars represent SD. Statistical significance was calculated using one-way ANOVA and post hoc Tukey’s test for multiple comparisons. *p < 0.05, **p < 0.005, ***p < 0.0005.

TCR signal strength is crucial in development of thymic Tregs (29), and the decrease in TCR signaling capacity in SKG mice results in a decrease in the proportion of thymic Tregs (19). Given that SKG *Ptpn22*^−^*^/^*^−^ mice had slightly improved signaling in DP thymocytes (Fig. 2A), we asked whether they had any changes in proportions of thymic Tregs. Both the percentages and the absolute cell numbers of FoxP3^+^ cells were similar in the thymuses of SKG *Ptpn22*^−^*^/^*^−^ and SKG mice (Fig. 5A), indicating comparable efficiency of selection of Tregs between these strains. The overall numbers of thymic Tregs in SKG were significantly reduced compared with control BALB/c mice (Fig. 5A), as reported previously (19).

Compared with BALB/c mice, numbers of T effector/memory cells and peripheral Tregs are increased in SKG mice, as the reduced generation of SP thymocytes results in a more lymphopenic peripheral environment (19). Comparison of Treg numbers in peripheral lymphoid organs of SKG *Ptpn22*^−^*^/^*^−^ and SKG mice showed that the former had a small, but significant increase in the numbers of CD25^+^FoxP3^+^ Tregs in spleen of adult animals (Fig. 5B). This change was confined to the spleen, as LNs from these mice showed comparable numbers of Tregs irrespective of *Ptpn22* status (Fig. 5C). We further assessed whether PTPN22 deficiency influenced Treg expansion upon mannan challenge. Compared with either untreated SKG or BALB/c control mice, increased proportions of Tregs were recovered from LNs and spleen in mannan-treated SKG and SKG *Ptpn22*^−^*^/^*^−^ mice (Fig. 5D). We noted a consistent, albeit not statistically significant, increase in the proportions of Tregs in the LN and spleen of SKG *Ptpn22*^−^*^/^*^−^ compared with SKG mice. Furthermore, a significant increase in the proportions of Tregs in the mesenteric LN, a key site of inducible Treg induction, was seen in SKG *Ptpn22*^−^*^/^*^−^ mice, suggesting that *Ptpn22* deficiency might enhance the propensity of naive T cells to differentiate into this lineage (Fig. 5D).

We tested the ability of SKG and SKG *Ptpn22*^−^*^/^*^−^ Tregs to suppress expansion of naive BALB/c T cells in vivo by cotransfer into empty Rag^−^*^/^*^−^ mice. In the absence of Tregs, the naive T cells expanded in the empty hosts, and the expansion was controlled to a similar extent by Tregs from either SKG or SKG *Ptpn22*^−^*^/^*^−^ mice (Fig. 5E). Therefore, on the SKG background, PTPN22-deficient Tregs are not more suppressive than SKG Tregs, in contrast to our previous findings that Tregs from C57BL/6 mice with normal ZAP70 expression are more potent suppressor cells when PTPN22 is absent (5). Given this result, we conclude that, although there was a slight increase in Treg numbers when PTPN22 was absent, it is unclear that this increase in numbers alone would have sufficient impact to account for the resistance to arthritis in this model.

### Effector SKG Ptpn22^−/−^ CD4 T cells have reduced propensity to polarize to the Th17 lineage

**FIGURE 6.**
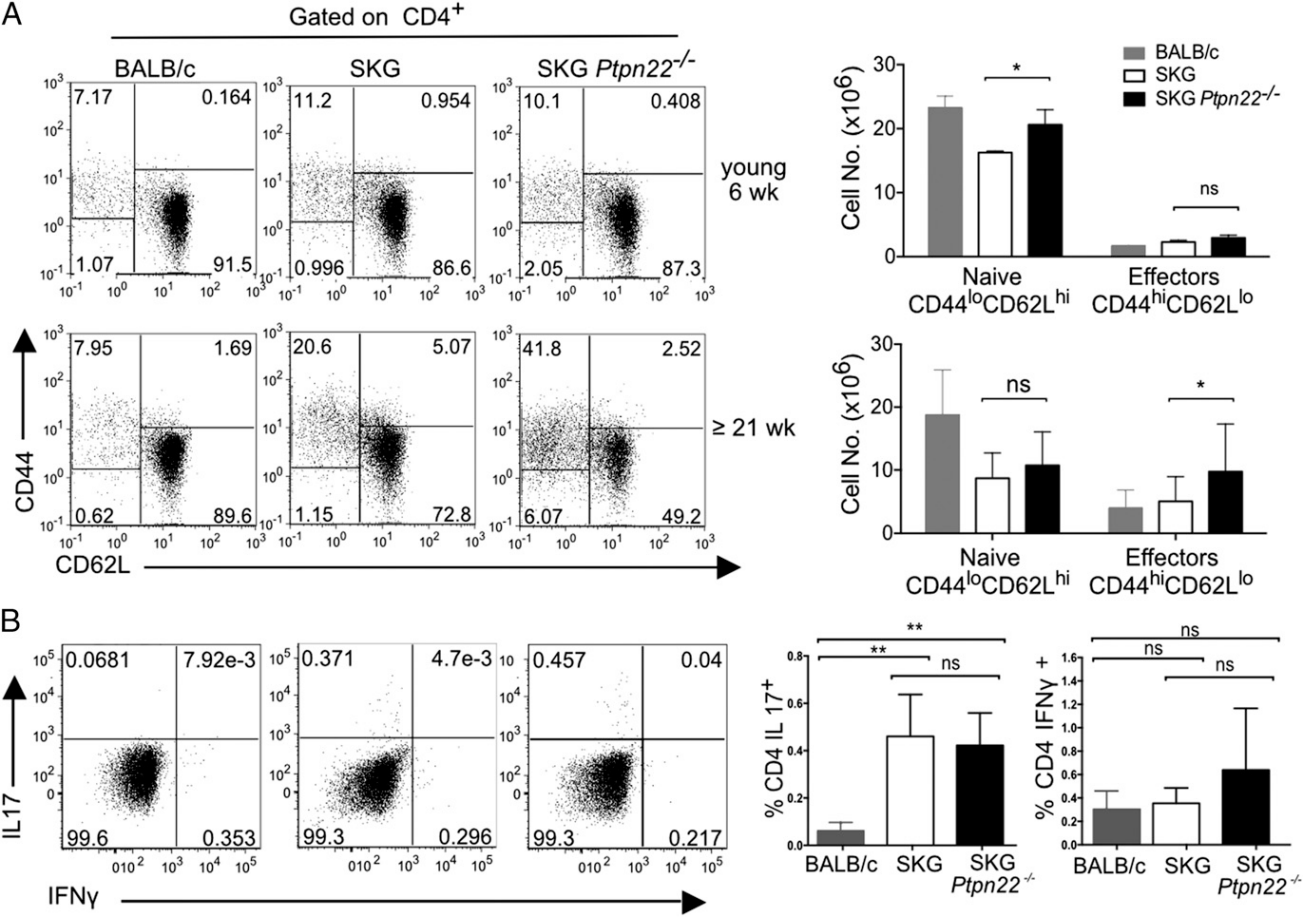
SKG *Ptpn22*^−^*^/^*^−^ mice have an expansion of CD4^+^ effector/memory T cells that increases with age. (**A**) Expression of CD44 and CD62L on gated CD4^+^ T cells to distinguish CD62L^hi^CD44^lo^ naive, and CD62L^lo^CD44^hi^ effector and memory T cells. Data shown were obtained from young (6-wk-old) and old (>21-wk-old) mice. Representative dot plots (*left*) and graphs with absolute cell numbers (*right*) from each genotype are shown. (**B**) Intracellular staining of IL-17 and IFN-γ in CD4^+^ LN T cells from older BALB/c, SKG, and SKG *Ptpn22*^−^*^/^*^−^ mice (>21 wk old) following stimulation with PDBu/ionomycin. A representative staining from three experimental repeats with minimum of three animals per groups is shown. Horizontal bars show the group means, and error bars represent the SD. Statistical significance calculated with one-way ANOVA and post hoc Tukey’s test, *p < 0.05, **p < 0.005.

On the B6 background, there is an expansion of *Ptpn22*^−^*^/^*^−^ T effector/memory cells with age (5, 6). Perturbations in T cell homeostasis are linked to onset of autoimmunity and are seen with autoimmune-prone PTPN22^R620W^-expressing individuals (30), so we asked whether there was equivalent expansion of effector cells in SKG mice or whether the SKG phenotype might suppress PTPN22-mediated expansion and thus explain the reduced arthritis in SKG *Ptpn22*^−^*^/^*^−^ mice. We analyzed the T cell phenotype of young (∼6 wk of age) and older (≥21 wk) at 6 wk and saw a small, but not statistically significant increase of effector cells (CD44^hi^ CD62L^lo^) on the SKG background compared with BALB/c and no difference between SKG and SKG *Ptpn22*^−^*^/^*^−^ mice. In older animals, SKG *Ptpn22*^−^*^/^*^−^ mice showed a clear expansion of effector cells, which was significantly greater than that seen in SKG mice, which in turn were significantly expanded compared with BALB/c mice (Fig. 6A). Analysis of the cytokine-producing potential of CD4 T cells from these animals ex vivo showed that, compared with BALB/c cells, both SKG and SKG *Ptpn22*^−^*^/^*^−^ CD4 T cells from LN had an increased, but similar capacity to produce IL-17, whereas the incidence of IFN-γ–producing cells was not significantly different between the strains (Fig. 6B). A higher incidence of IL-17–producing cells has been noted for SKG mice (25), although the percentage of spontaneous Th17^+^ cells is considerably lower in our animal facility than previously published, presumably reflecting different exposure to commensal microbiota.

**FIGURE 7.**
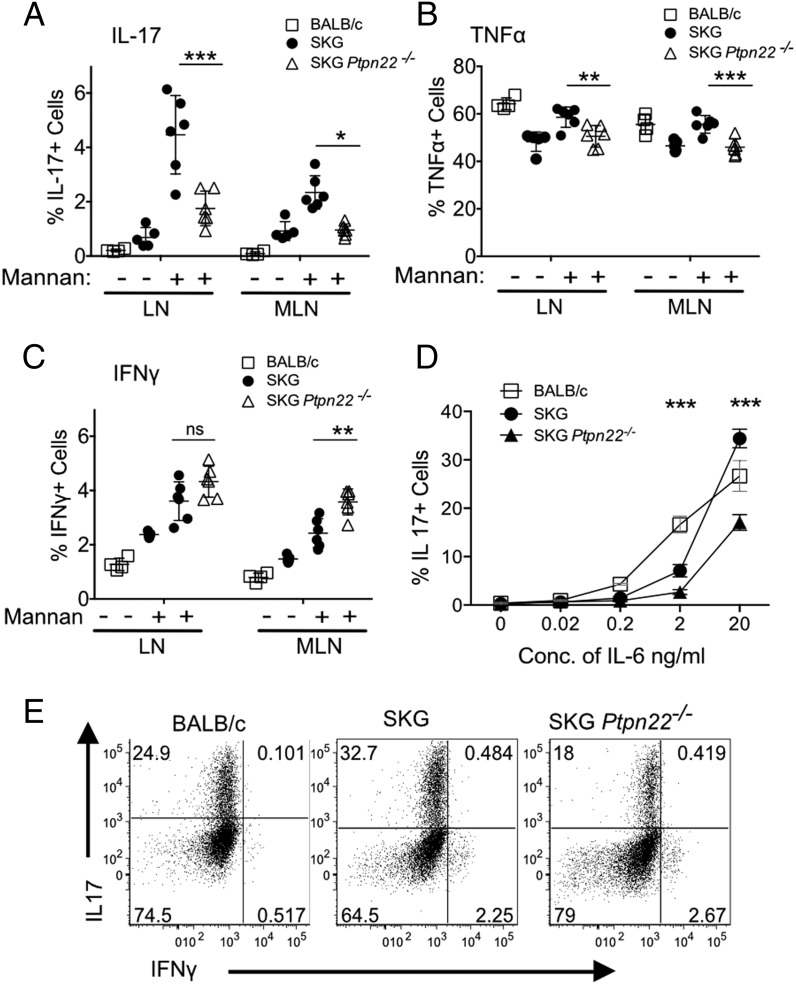
Reduced polarization of IL-17 CD4^+^ T cells in vivo and in vitro in SKG *Ptpn22*^−^*^/^*^−^ mice. (**A**–**C**) Quantification of the percentage of CD4^+^ T cells in peripheral draining (axial, inguinal, brachial, and popliteal) and mesenteric LN from BALB/c, SKG, and SKG *Ptpn22*^−^*^/^*^−^ mice that were positive for intracellular staining of IL-17, TNF-α, and IFN-γ following stimulation with PDBu/ionomycin after 3 wk of mannan challenge. Each symbol represents individual animals. One representative staining of three experimental repeats with minimum of three animals per groups is shown. (**D** and **E**) FACS-sorted naive (CD4^+^CD25^−^CD45RB^hi^) cells from 6-wk-old BALB/c, SKG, and SKG *Ptpn22*^−^*^/^*^−^ mice were activated with plate-bound anti-CD3 and anti-CD28 in the presence of TGF-β, IL-1β, IL-23, and varying amounts of IL-6 for 3 d, and then restimulated with PDBu/ionomycin for 4 h in presence of brefeldin A. (D) Quantitation of IL-17^+^ cells after culture with varying doses of IL-6, and (E) FACS dot plots are shown for IL-17 and IFN-γ expression with 20 ng/ml IL-6. Statistical significance calculated with two-way ANOVA; *p < 0.05, **p < 0.005, ***p < 0.0005.

Development of arthritis in SKG mice is dependent on IL-17–producing arthritogenic Th17 cells, which are promoted by IL-6 secreted predominately by APCs (31). Deficiency of either IL-6 or IL-17 completely inhibits the arthritis, whereas lack of IFN-γ can exacerbate disease in SPF mice (31, 32). As SKG *Ptpn22*^−^*^/^*^−^ mice developed less severe arthritis than SKG mice, we compared cytokine production in peripheral CD4^+^ T cells in these animals following injection of mannan and induction of arthritis. Peripheral and mesenteric LNs were isolated separately from mice 14 d after mannan injection and stained for cytokine production after a brief stimulation with PDBu and ionomycin. Production of key cytokines IL-17 and TNF-α (Fig. 7A, 7B) was significantly reduced in SKG *Ptpn22*^−^*^/^*^−^ mice following arthritis induction compared with SKG mice. IL-17–producing cells from both mouse strains were CD4^+^, αβ-TCR^+^, and CCR6^+^, indicating they were conventional Th17 cells (data not shown). There was a tendency of SKG *Ptpn22*^−^*^/^*^−^ mice to produce more IFN-γ–secreting cells (Fig. 7C); however, this was significant only in the mesenteric LNs. To ascertain whether the reduced tendency of cells from SKG *Ptpn22*^−^*^/^*^−^ to differentiate to the IL-17 lineage was cell intrinsic or a consequence of the cytokine milieu, we differentiated purified naive CD4^+^ T cells from SKG and SKG *Ptpn22*^−^*^/^*^−^ mice in vitro in the presence of increasing concentrations of IL-6 together with TGF-β. Significantly fewer IL-17–producing cells were generated from SKG *Ptpn22*^−^*^/^*^−^ CD4 T cells than from SKG mice (Fig. 7D, 7E), suggesting that loss of PTPN22 produces an inherent bias against polarization to the IL-17 lineage that may contribute to the reduction in susceptibility to arthritis observed in this strain of mice.

## Discussion

In this study, we examined the influence of the phosphatase PTPN22 on the development of autoimmune disease in combination with an established model of autoimmune arthritis, the SKG mouse. The SKG mouse develops arthritis as a consequence of having a reduction in functional ZAP70 protein, and given that ZAP70 is both a direct target of PTPN22 and a target of the Src-family kinase, Lck, whose activity is regulated by PTPN22, we anticipated that the combined mutations might exacerbate development of disease. On the contrary, we found that SKG mice lacking PTPN22 had both lower incidence and decreased severity of arthritis following induction of disease by administration of mannan.

Susceptibility to autoimmune diseases involves complex interactions between genetics and environmental factors. The major genetic contribution to diseases such as arthritis is the MHC (33), but recent genome-wide association studies have identified a number of other contributing genes, the most predominant of which is *PTPN22* (10). PTPN22 is present in all hematopoietic cells (34), and the major disease-associated polymorphism, *PTPN22-C1858T*, has been shown to affect myeloid and B cell, as well as T cell, function (2). The SKG arthritic mouse is a good model for the human RA, as joint inflammation and damage mirror that found in patients (24). Similar to humans, arthritic disease in SKG mice is triggered by environmental factors. Sakaguchi and colleagues (31) showed that subclinical fungal infections drove the inflammatory signals that instigated spontaneous arthritis, an effect that was recapitulated by direct administration of fungal products, such as zymosan or mannan, to SPF-bred SKG mice. These fungal products induce IL-6 production, which in turn drives differentiation of autoreactive T cells to the Th17 lineage. In the context of a more self-reactive TCR repertoire, which differentiates in the presence of the mutant ZAP70 alleles (11), SKG Th17 CD4 T cells migrate to the joints causing the characteristic tissue damage.

The *PTPN22^C1858T^* mutation in human cells of the myeloid lineage has been shown to interfere with upregulation of type 1 IFNs after TLR4 stimulation by LPS, and mouse *Ptpn22*^−^*^/^*^−^ bone marrow–derived macrophages showed a similar defect (35, 36). This reduction in type 1 IFNs may impede their tissue-protective effects in the presence of inflammatory cytokines and thus contribute to autoimmune disease susceptibility in humans. In contrast, production of proinflammatory cytokines, TNF-α and IL-6, was shown to be equivalent to WT cells following TLR4 stimulation for either mouse *Ptpn22*^−^*^/^*^−^ or human *PTPN22^C1858T^* myeloid cells (36). Given the central role of macrophage-derived IL-6 in driving autoimmune disease in SKG mice (25), we confirmed that SKG *Ptpn22*^−^*^/^*^−^ peritoneal macrophages produced equivalent levels of IL-6 to SKG macrophages in response to mannan stimulation. Mannan is a prototypic activator of the lectin pathway of complement activation, and incidence of disease is suppressed in SKG mice lacking the C5a receptor (25); our data would argue against an involvement of PTPN22 in this signaling pathway.

Deleting PTPN22 expression on the SKG background was predicted to increase signaling through the TCR and potentially worsen disease. Indeed, we showed that peripheral SKG *Ptpn22*^−^*^/^*^−^ CD4 T cells were more responsive to TCR stimulation, but with the consequence that they produced more IL-2 than SKG T cells. The production of IL-2 is not only important for Treg development and maintenance, but has also been reported to inhibit Th17 generation, as blockage of IL-2 or deletion of STAT-5 leads to enhanced production of Th17 cells (37). Therefore, the change in cytokine balance in SKG *Ptpn22*^−^*^/^*^−^ mice was likely to influence disease progression. That PTPN22-deficient T cells produce more IL-2 than WT cells upon stimulation has been shown in mice previously (6), and it is interesting in this regard, that a study comparing cytokine responses by CD4 T cells from individuals expressing the disease-associated PTPN22^620WW^ protein also showed a bias toward Th1 responses with more production of IL-2 and IFN-γ and reduced production of IL-17, in comparison with T cells expressing PTPN22^620RR^ (38). Together these results indicate that signaling through PTPN22 can influence cytokine production in T cells expressing WT ZAP70 in addition to the SKG mutation, and that this may influence susceptibility to autoimmune disease.

Peripheral tolerance is maintained in vivo by keeping an appropriate balance between self-reactive T cells and Tregs (39), and *Ptpn22* deficiency has been shown to be protective in NOD and experimental autoimmune encephalomyelitis mice model of disease due to increased Treg numbers (28, 40). Surprisingly, however, with the exception of spleen, we did not see a consistent expansion of Tregs in lymphoid tissue of SKG *Ptpn22*^−^*^/^*^−^ older mice beyond that seen in SKG mice, despite the former having almost twice as many CD44^+^ effector T cells, nor did we find any increased potency in suppressive ability between Tregs from SKG and SKG *Ptpn22*^−^*^/^*^−^ mice. These results are in contrast to a study in which the SKG mutation was combined with a deletion of the negative regulator SLAP (41). SKG SLAP^−^*^/^*^−^ mice also showed reduced susceptibility to induction of arthritis, but, in this case, there was a clear increase in both natural Tregs in the thymus and induced Tregs in the periphery of SKG SLAP^−^*^/^*^−^ mice, which were further expanded after injection of zymosan. In contrast to the SKG SLAP^−^*^/^*^−^ mutant mice, where there was ∼300-fold increase in Treg numbers, the increase of Tregs in SKG *Ptpn22*^−^*^/^*^−^ mice was only ∼20% above that found in SKG mice and not observed consistently in all lymphoid organs.

Although the SKG mutation is a robust predisposition allele for development of arthritis following innate stimulation, it is clear that in this model the tipping point between generation of pathogenic T cells and maintenance of tolerance is finely balanced. To date any modulation of precisely SKG levels of ZAP70 function has tended to reduce susceptibility to arthritis induction. As described above, combining SKG with SLAP knockout diminished arthritis by improving Treg numbers (41). In another model in which ZAP70 function is compromised, the knock-in mouse ZAP70^YYAA^ in which Tyr residues 315 and 319 are substituted with Ala, ZAP70 function was also considerably impaired compared with WT ZAP70 (20). ZAP70^YYAA^ generated rheumatoid factor following zymosan injection, but the mice were very resistant to the development of arthritis. Ca^2+^ flux in DP thymocytes and responses of peripheral ZAP70^YYAA^ T cells were intermediate between SKG and WT, similar to the levels we reported in this work with SKG Ptpn22^−^*^/^*^−^ mutant T cells. Yet ZAP70^YYAA^ thymocytes showed improved deletion of Mtv-reactive TCR Vβ chains, which we did not observe in SKG *Ptpn22*^−^*^/^*^−^ thymocytes. ZAP70^YYAA^ T cells were similar to SKG *Ptpn22*^−^*^/^*^−^ T cells in that they produced more IL-2 and were slightly less efficient at differentiating to the Th17 lineage than SKG T cells, which, together with improved thymic deletion, gave near-complete protection against disease. In the case of SKG *Ptpn22*^−^*^/^*^−^ mice, incidence of arthritis was reduced, but not as profoundly suppressed as in ZAP70^YYAA^ mice, consistent with protection stemming from a combination of a reduction in Th17 effector cell generation together with a slight improvement in Treg generation rather than a change in repertoire selection.

The contribution of the *PTPN22^C1858T^* variant to RA susceptibility in humans is multifactorial, as this mutation affects several hematopoietic lineages: T cells, B cells, and myeloid cells, all of which have been shown to contribute to pathogenicity in RA (42). Disease in the SKG mouse model is thought to originate primarily from an altered TCR repertoire due to a shift in thymic selection toward specificities with higher avidity for self-peptide/MHC, which, when combined with infection by pathogens, leads to induction of RA, and similar mechanisms may contribute to human disease (43). In this study, we showed that, whereas loss of PTPN22 improved the activity of mutant ZAP70^skg/skg^, this had little consequence for selection events in the thymus, and yet significantly reduced the development of arthritis following mannan injection. Our studies suggest that the improved incidence and severity of disease were mainly due to an intrinsic bias of SKG *Ptpn22*^−^*^/^*^−^ T cells to change their cytokine profile upon stimulation, with a reduction in the frequency of disease-inducing IL-17 cells and a trend toward production of IFN-γ instead. Combined with a slight increase in Treg numbers, the reduction in IL-17 production was sufficient to ameliorate disease severity, thus illustrating how even very small alterations in T cell signaling may have profound consequences for the maintenance of T cell tolerance.

## Supplementary Material

Data Supplement
