# Supplementary material for: Loss of the Protein Tyrosine Phosphatase PTPN22 Reduces Mannan-Induced Autoimmune Arthritis in SKG Mice
Source: J Immunol. 2016 Jun 10;197(2):429–40. doi: 10.4049/jimmunol.1502656 (PMC4932175; doi:10.4049/jimmunol.1502656)
Supplement: Data Supplement [file JI_1502656.zip › JI_1502656_Supplemental_Figure_1.pdf]

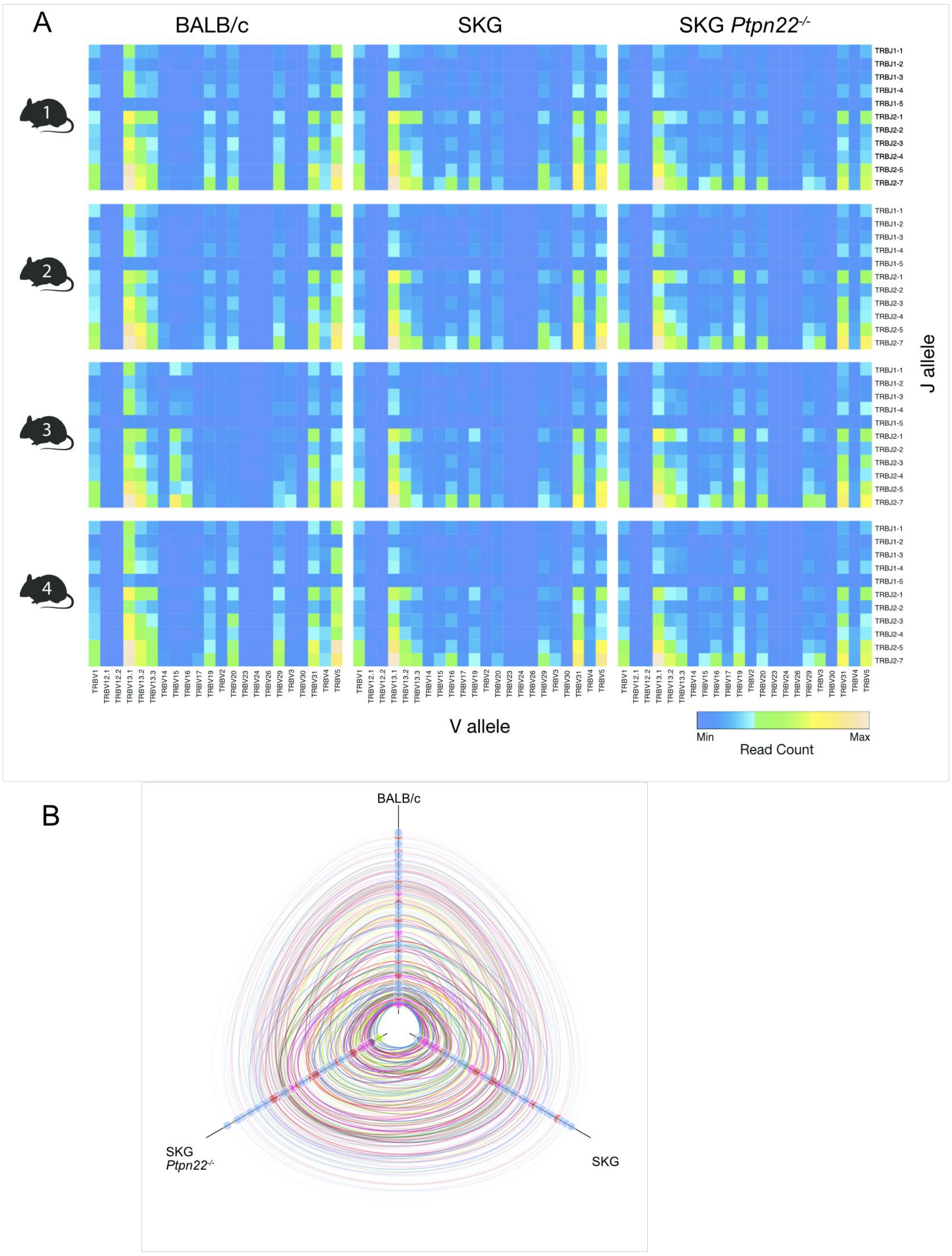

Supplementary Figure 1. TCRβ CDR3 analysis shows similar repertoires between SKG and SKG Ptpn22$^{-/-}$ mice. (A) Heatmaps of relative frequencies of V and J segment usage combinations. Colour indicates the proportion of the total number of reads that are composed of each V and J allelic combination. Each panel represents the TCRB VJ combaintions from a single mouse, grouped into columns based on mouse genotype, as indicated in the column header text. (B) Radial plot of median percentage of reads mapping to individual VJ combinations for each mouse genotype. Circular nodes located on each radial axis indicate the common logarithm of the percentage of the repertoire comprised of each VJ combination. More highly represented VJ combinations are distal, and less represented combinations are towards the centre of the chart (maximum value 3.35%). Nodes representing the same VJ combination are connected by lines and the opacity of each line is scaled according to the coefficient of variation for that VJ combination to highlight clones with the highest degree of variation.
